# Extracts of Strawberry Fruits Induce Intrinsic Pathway of Apoptosis in Breast Cancer Cells and Inhibits Tumor Progression in Mice

**DOI:** 10.1371/journal.pone.0047021

**Published:** 2012-10-10

**Authors:** Ranganatha R. Somasagara, Mahesh Hegde, Kishore K. Chiruvella, Anjaneyulu Musini, Bibha Choudhary, Sathees C. Raghavan

**Affiliations:** 1 Department of Biochemistry, Indian Institute of Science, Bangalore, Karnataka, India; 2 Institute of Bioinformatics and Applied Biotechnology (IBAB), Bangalore, Karnataka, India; Wayne State University School of Medicine, United States of America

## Abstract

**Background:**

The consumption of berry fruits, including strawberries, has been suggested to have beneficial effects against oxidative stress mediated diseases. Berries contain multiple phenolic compounds and secondary metabolites that contribute to their biological properties.

**Methodology/Principal Findings:**

Current study investigates the anticancer activity of the methanolic extract of strawberry (MESB) fruits in leukaemia (CEM) and breast cancer (T47D) cell lines *ex vivo,* and its cancer therapeutic and chemopreventive potential in mice models. Results of MTT, trypan blue and LDH assays suggested that MESB can induce cytotoxicity in cancer cells, irrespective of origin, in a concentration- and time-dependent manner. Treatment of mice bearing breast adenocarcinoma with MESB blocked the proliferation of tumor cells in a time-dependent manner and resulted in extended life span. Histological and immunohistochemical studies suggest that MESB treatment affected tumor cell proliferation by activating apoptosis and did not result in any side effects. Finally, we show that MESB can induce intrinsic pathway of apoptosis by activating p73 in breast cancer cells, when tumor suppressor gene p53 is mutated.

**Conclusions/Significance:**

The present study reveals that strawberry fruits possess both cancer preventive and therapeutic values and we discuss the mechanism by which it is achieved.

## Introduction

A diet rich in fruits and vegetables has been associated with a reduced risk of diseases, such as cardiovascular disorders and cancer [1], [2], [3], [4]. Previously, an efficient food based approach for cancer prevention was studied in a rodent model of colon carcinoma [5]. It has been shown that the phytochemicals present in fruits and vegetables are more effective than their individual constituents in preventing cancer through both additive and synergetic effects [6], [7]. Hence, it is important to study the potential activity of fruits and vegetables using whole extracts containing various phytochemicals, instead of using purified molecules or fractions enriched with certain classes of molecules.

Previous studies suggest that consumption of berry fruits can have beneficial effects against diseases such as cancer [8]. Berries contain multiple phenolic compounds, which contribute to their biological properties. It has been suggested that bioactive components of berry invoke anti-cancer effects through various complementary and overlapping mechanisms of action including the induction of metabolizing enzymes, modulation of gene expression etc. However, their definitive mechanism of action is largely unknown [9].

Strawberries are a good source of natural antioxidants [10], which can be linked to the level of phenolic compounds in these fruits [11]. A recent study showed that strawberry extracts exhibit a higher level of antioxidant capacity against free radical species including superoxide radicals, hydrogen peroxide, hydroxyl radicals, and singlet oxygen [12]. Strawberries contain antioxidants, such as vitamin C, hydroxycinnamic acids, anthocyanins and flavonoids [11], [13]. Besides, due to relatively high content of ellagic acid, an antioxidant that can exert antimutagenic and anticarcinogenic effect, it has been a preferred target for cancer studies [14], [15]. A study has also shown that strawberries have potent anti-proliferative activity on human liver cancer cells, HepG2 [16]. However, there are no studies to investigate its anticancer potential and the mechanism by which it exerts its effect.

In most of the cancers, mutation in the tumor suppressor gene, p53, significantly contributes to cancer development [17]. Hence, p53 analogues like p73, p63 etc. are shown to play a similar function during oncogenesis [18]. p73 shares significant sequence as well as functional homology with p53. The central specific DNA binding sequence, N-terminal activation and C-terminal oligomerization domains share significant sequence homology between them. Similar to p53, proteins like BAX, PUMA are also direct targets of p73 [19].

Various phytochemicals and chemically synthesized small molecules induce apoptosis, largely through the activation of intrinsic pathway. Intrinsic apoptotic pathway involves a variety of stimuli from inside the cells like DNA damage, ROS generation etc. The major players of this pathway include BCL2 family of proteins, which are mainly classified as proapoptotic and antiapoptotic proteins, based on their activity. An imbalance in the ratio between these classes of proteins leads to damage of mitochondrial membrane integrity resulting in CYTOCHROME C release and CASPASE 9 followed by CASPASE 3 activation [20].

In the present study, we show that extracts prepared from Indian strawberry fruits induce cytotoxicity by activating intrinsic pathway of apoptosis, through a p53 independent mechanism in breast cancer cells. MESB also interferes with progression of tumors in breast cancer mouse models and results in the extended lifespan without affecting other cellular functions and body weight. Most importantly, we also provide evidence that strawberry consumption can delay tumorigenesis in mice.

**Figure 1:**
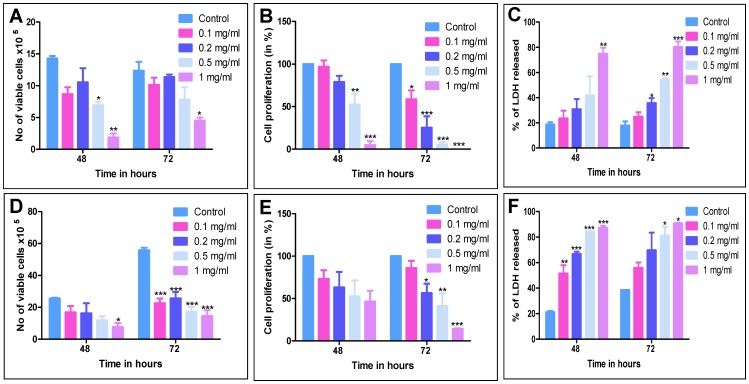
Assessment of MESB induced cytotoxicity in leukemic (CEM) and breast cancer (T47D) cell lines. CEM and T47D cells were treated with increasing concentrations of MESB (0.1, 0.2, 0.5 and 1 mg/ml) and cells were harvested after 48 or 72 h of treatment and subjected to trypan blue, MTT and LDH assays. **A.** Determination of cell viability by trypan blue assay in CEM cells. **B.** Evaluation of cell proliferation by MTT assay in CEM cells. **C.** Bar diagram showing release of lactate dehydrogenase following MESB treatment in CEM cells. **D.** Assessment of cell viability using trypan blue assay in T47D cells. **E.** Determination of cell proliferation by MTT assay in T47D cells after MESB treatment. **F.** LDH assay showing release of lactate dehydrogenase in T47D cells following addition of MESB. In each panel, error bars were calculated based on results obtained from minimum of three independent experiments. In all panels, *p<0.05, **p<0.005, and ***p<0.0005.

## Materials and Methods

### Chemicals and Reagents

All chemicals used in the present study were of analytical grade and purchased from Sigma-Aldrich (USA) and antibodies were purchased from Santa Cruz Biotechnology (USA) and Cell Signalling Technology (USA).

### Preparation of Methanolic Extract of Strawberry (MESB)

Indian strawberry fruits were purchased from the local markets, cut into small pieces and dried in shadow. The powdered strawberry was then extracted with methanol. Following evaporation, crude methanolic extracts were stored at room temperature under sterile conditions until further use.

### Cell Culture

Human T-cell leukemia cells, CEM and human breast cancer cells, T47D were purchased from National Centre for Cell Science, Pune (India). Cells were cultured in RPMI 1640 (Sera Lab, UK) containing 10% FBS (Gibco BRL, USA), 100 U of Penicillin G/ml and 100 µg of streptomycin/ml (Sigma-Aldrich, USA) at 37°C in a humidified atmosphere containing 5% CO_2_.

**Figure 2:**
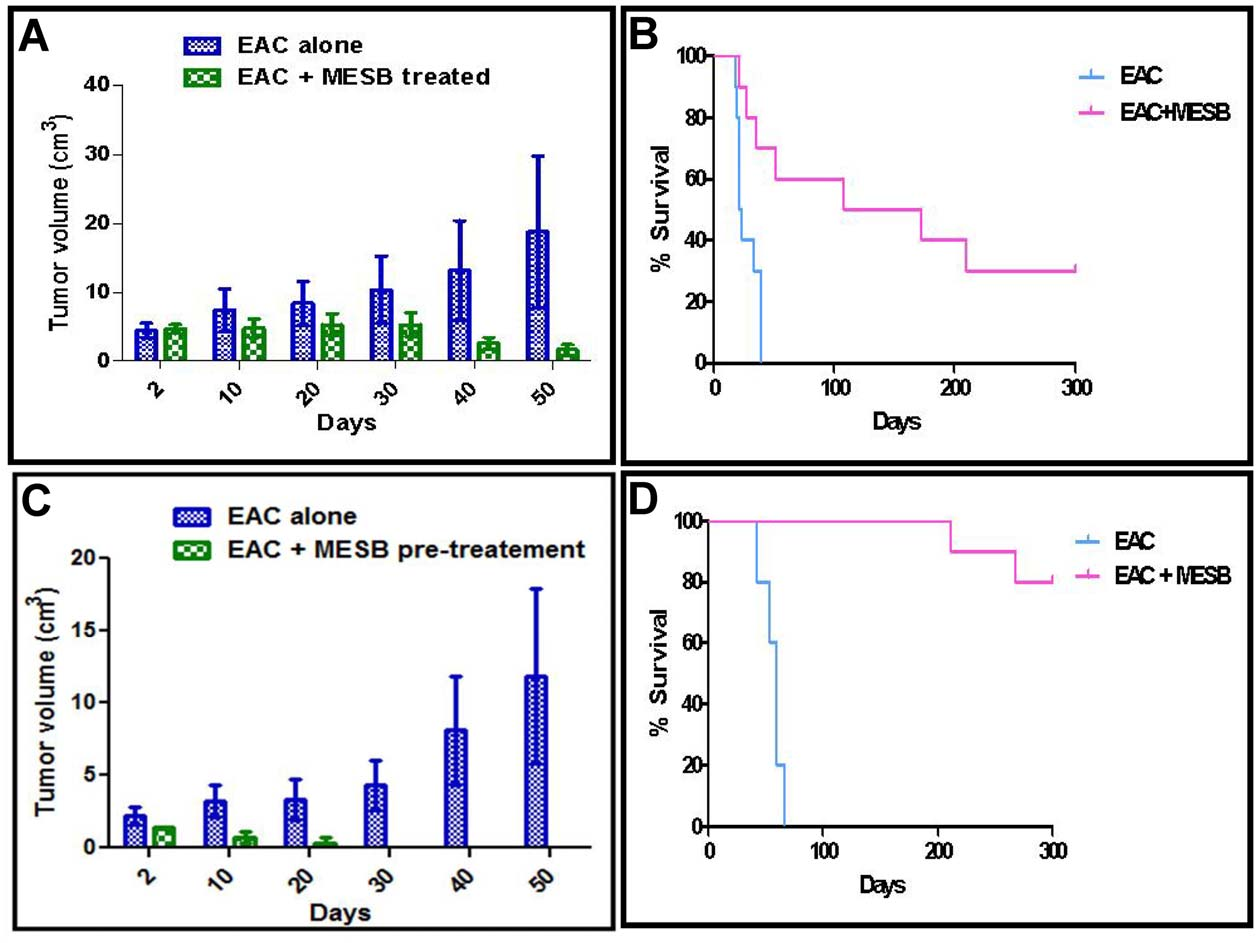
Effect of MESB on progression of tumor in mice. Solid tumor was induced in Swiss albino mice by injecting breast adenocarcinoma cells. Following tumor development, mice were orally treated with MESB (2 g/kg body weight) until 45^th^ day. **A.** Effect of MESB on tumor progression at different time points. Data shown is derived from three independent experiments containing 10 animals each. Error bars indicate standard deviation. **B.** Kaplan-Meier survival curves of mice treated with MESB. **C.** The chemopreventive effect of MESB on EAC induced mice. The experimental mice were orally fed with MESB for 20 days prior to the EAC injection and were compared with control group, which did not receive pretreatment by MESB. **D.** Kaplan-Meier survival curves of mice pretreated with MESB.

### Trypan Blue Dye Exclusion Assay

The effect of MESB on cell viability of CEM and T47D cells was determined by trypan blue dye exclusion assay [21], [22]. CEM and T47D cells were cultured (0.75×10^5^ cells/ml) and increasing concentrations (0.1, 0.2, 0.5 and 1 mg/ml) of MESB was added and incubated. Cells were collected after 48 and 72 h of incubation. Number of viable cells was determined by trypan blue staining. Experiments were repeated three independent times and the data was presented as bar diagram with error bars.

### MTT Assay

The MTT assay was performed as described earlier [22], [23]. Both CEM and T47D cells (0.75×10^5^ cells/ml) were treated with MESB (0.1, 0.2, 0.5 and 1 mg/ml) and incubated for 48 and 72 h. Cells were collected and subjected to MTT assay. Experiments were repeated three independent times, each with duplicate reactions and presented as bar diagram with error bars.

### LDH Release Assay

Lactate dehydrogenase (LDH) assay was performed to assess the LDH release into the media following MESB treatment (0.1, 0.2, 0.5 and 1 mg/ml) on both CEM and T47D cells after 48 and 72 h of incubation as described earlier [23]. The cells were lysed using 0.1% Triton-X 100 in PBS. The amount of LDH released in both culture media and cell lysate was measured at 490 nm using an ELISA reader (BioRad, USA). The percentage of LDH release was calculated as LDH release in media/(LDH release in media + intracellular LDH release) × 100.

**Figure 3:**
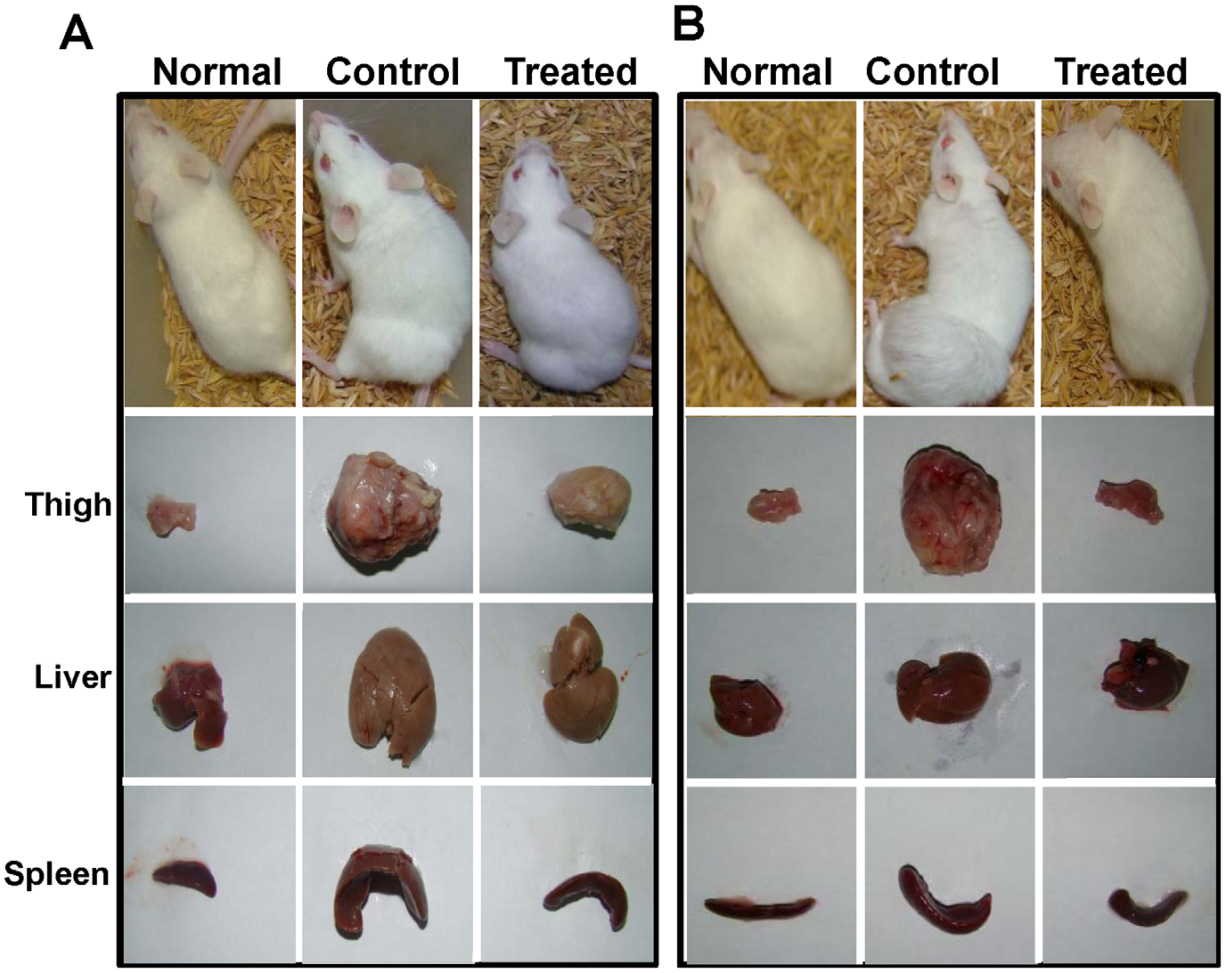
Gross appearance of mice and its organs following MESB treatment. Gross appearance of mice and its selected organs following treatment with MESB on tumor bearing mice after 30^th^ **(A)** and 45^th^ **(B)** days of tumor development.

### Western Blot Analysis

Cell lysate was prepared following treatment with MESB on T47D cells (0, 0.1, 0.4, 0.7 mg/ml for 48 h) as described and used for western blotting [24]. Western blotting experiments were performed by using ∼30 µg of protein. Samples were electrophoresed on 8-12% SDS-PAGE, proteins were transferred to PVDF membrane (Millipore, USA) and probed with respective primary and secondary antibodies. The primary antibodies against MCL-1, BCL-xL, BAX, BID, p53, MDM2, p73, PARP1, SMAC/DIABLO, CYTOCHROME C, APAF1, CASPASE 3 and CASPASE 9 were used. Anti-TUBULIN and anti-ACTIN were used as the loading control. The blots were developed using chemiluminescent reagent (Immobilon™ western, Millipore, India) and scanned using gel documentation system (LAS 3000, FUJI, JAPAN).

Western blotting studies were also performed using cytosolic extracts prepared following treatment with MESB. T47D cells were treated with MESB for 48 h (0, 0.1, 0.4, 0.7 mg/ml). Cytosolic fractions were separated using mitochondrial extraction kit (IMGENEX, Cat.No. 10082k), western blotting was performed using anti-CYTOCHROME C and anti-SMAC/DIABLO.

**Figure 4:**
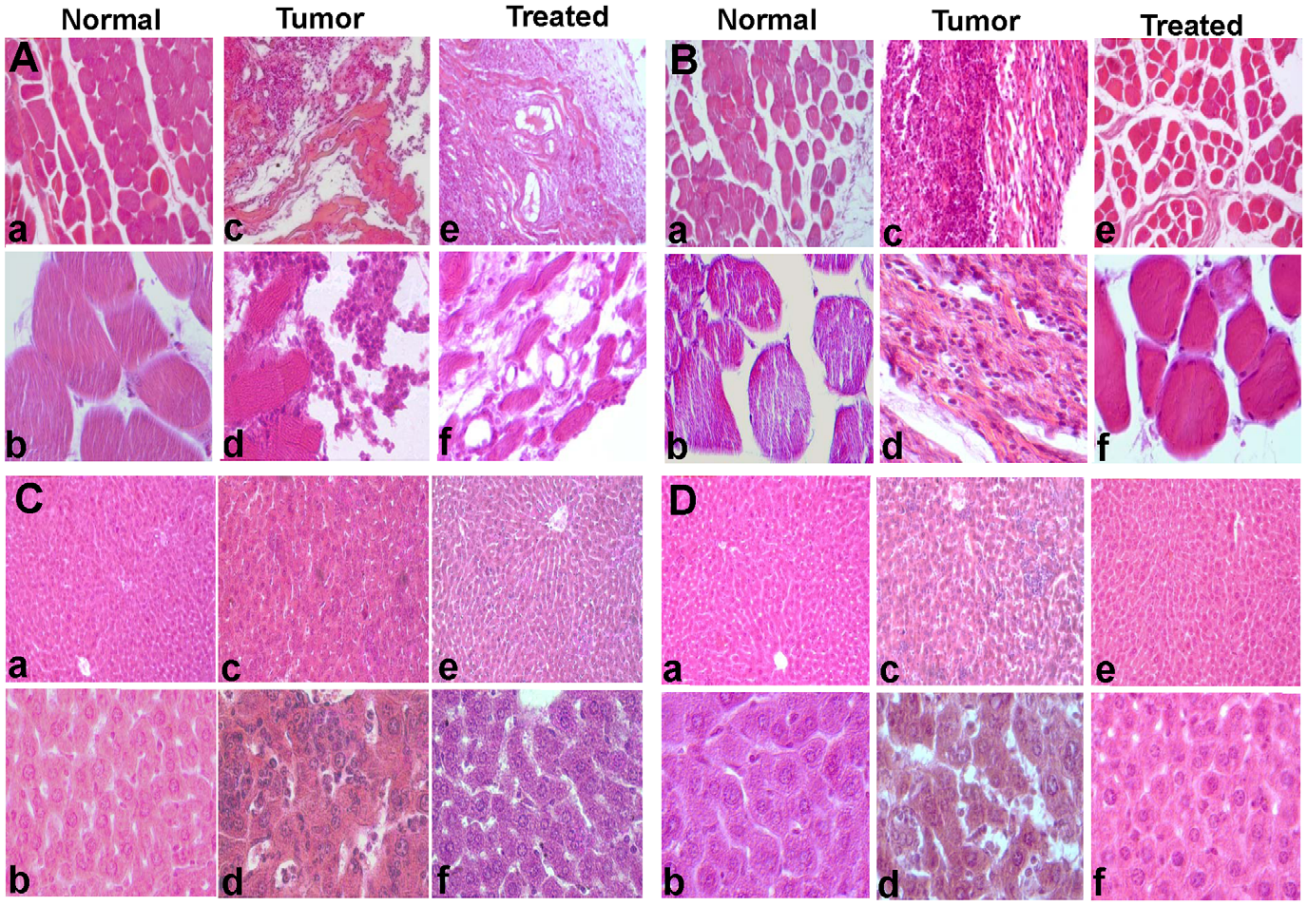
Histopathology of the tumor tissues and liver of mice following MESB treatment. Histopathalogical sections of thigh and liver of a tumor bearing mouse with and without treatment with MESB after 30^th^ day A(a–f) and C(a–f) and 45^th^ day B(a–f) and D(a–f) of development of tumor. Magnification shown are 10x (a, c and e in all panels) and 40x (b, d and f in all panels).

### Animals and Ethics Statement

Mice were maintained as per the principles and guidelines of the ethical committee for animal care of Indian Institute of Science (IISc) in accordance with Indian National Law on animal care and use. The experimental design of the present study was approved by Institutional animal ethics committee (Ref. CAF/Ethics/125/2007/560), Indian Institute of Science, Bangalore, India.

Swiss albino mice, 6–8 weeks old weighing approximately 18–22 g were purchased from central animal facility, Indian Institute of Science, Bangalore, India and maintained in the animal house, Department of Biochemistry, IISc. The animals were housed in polypropylene cages and provided standard pellet diet (Agro Corporation Pvt. Ltd., India) and water ad libitum. The standard pellet diet composed of 21% protein, 5% lipids, 4% crude fiber, 8% ash, 1% calcium, 0.6% phosphorus, 3.4% glucose, 2% vitamin and 55% nitrogen-free extract (carbohydrates). The mice were maintained under controlled temperature and humidity with a 12 h light/dark cycle.

**Figure 5:**
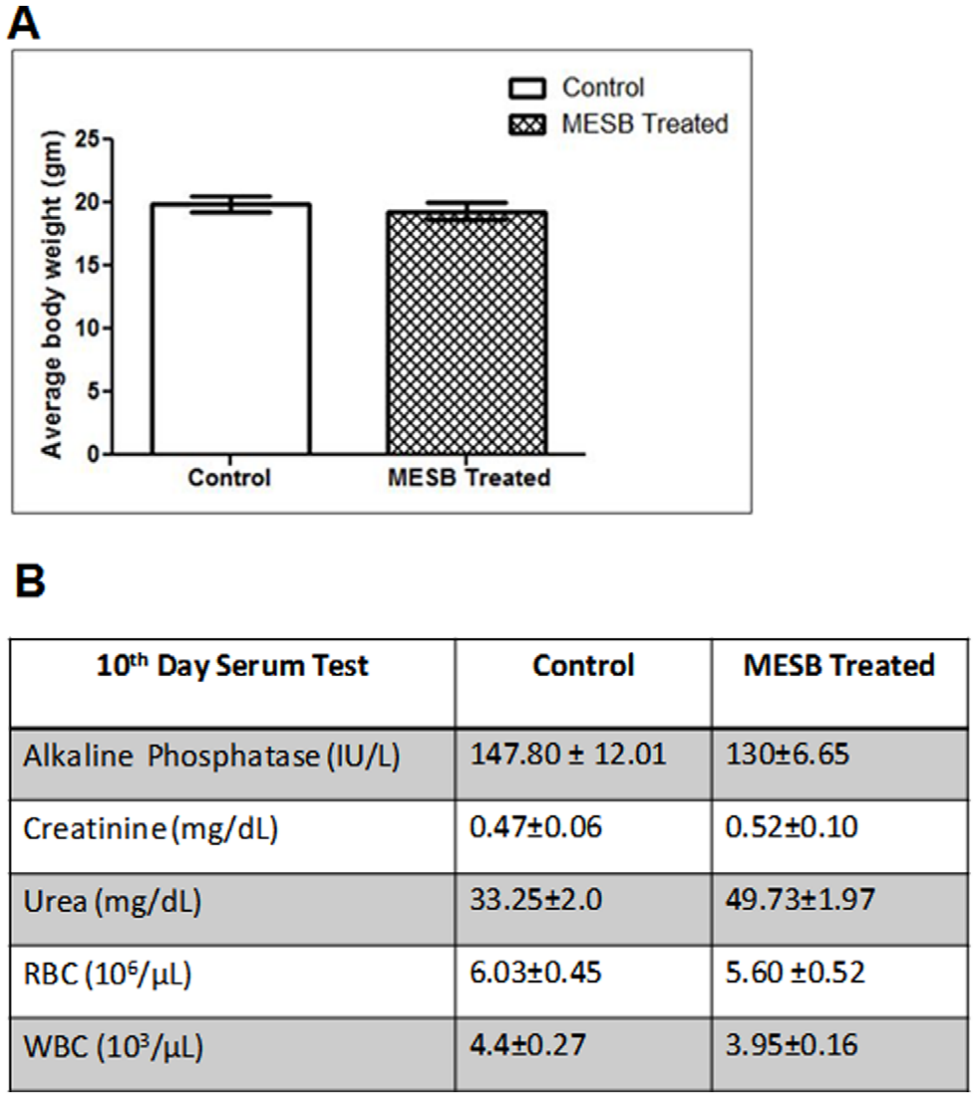
Evaluation of side effects of MESB in mice. Mice were orally fed with MESB (2 g/kg. b. wt) for 10 days. **A.** Data showing average body weight changes in the control (n = 8) and MESB treated mice (n = 8). In all the cases, error bars are indicated. **B.** Hematological and serum profile of mice following oral feeding with MESB at day 10 (n = 8 for both control and treated). Values are indicated in mean ± SEM.

### Preparation of Breast Adenocarcinoma Cells

Ehrlich ascites carcinoma (EAC) is an undifferentiated carcinoma, originally hyperdiploid and has high transplantable capability, rapid proliferation, shorter life span and 100% malignancy. EAC resembles human tumors which are most sensitive to chemotherapy due to the fact that they are undifferentiated and have a rapid growth rate. A fixed number of viable breast adenocarcinoma cells (1×10^6^ cells/22 g b. wt) were implanted into the peritoneal cavity of each donor mouse and allowed to multiply. The tumor cells were withdrawn, diluted in saline, counted and re-injected (1×10^6^ cells/animal) to the right thigh of experimental animal for development of solid tumor as described [25].

**Figure 6:**
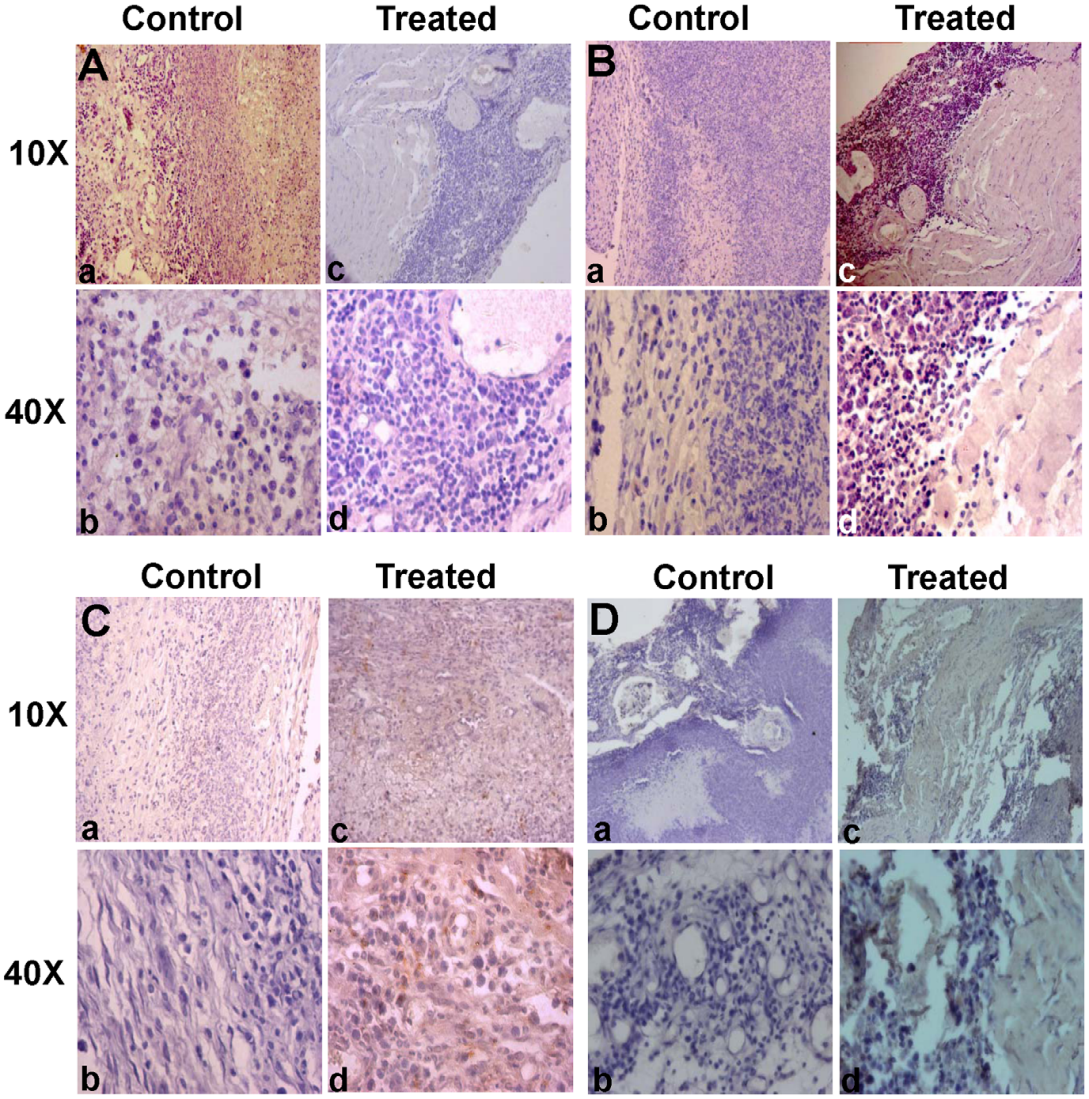
Immunostaining studies for apoptotic and DNA damage markers following treatment of mice bearing tumors with MESB. A-D. Ki67, p53BP1, BID and t-BID immunostaining of untreated (a, b) and treated (c, d) tumor tissues (30^th^ day of treatment). Antibodies used were Ki67 (**A**) p53BP1 (**B**), BID (**C**) and t-BID (**D**).

### Evaluation of Antitumor Activity of MESB in Mouse

In each experiment, out of 30 Swiss albino mice, 20 were injected with Ehrlich ascites carcinoma cells for developing the solid tumor. Group 1 served as untreated (normal) control (n = 10). EAC injected animals were divided into 2 groups of 10 animals each. Group 2 was considered as tumor control and received no treatment. Group 3 received oral feeding of 2 g/kg of MESB dissolved in water after 12 days of tumor development and continued up to 45 days. The experiment was repeated 3 independent times.

Size of the developing tumor was measured in both group 2 and 3 animals by using vernier callipers on alternative days for the entire life span of the animals. Tumor volume was calculated using the formula V = 0.5ab^2^, where ‘a’ and ‘b’ indicates the major and minor diameter, respectively [25], [26]. At the end of 30^th^ and 45^th^ day of experimental period, one animal from each group [normal (group 1), tumor (group 2) and MESB treated tumor animals (group 3)] was sacrificed by cervical dislocation, tissues were collected and stored under appropriate conditions. Each experiment was repeated three independent times.

The percentage of increase in lifespan was calculated and compared with control animals. The death pattern for control animals and MESB treated animals was recorded and % increase in lifespan was calculated using the formula [(T-C)/C] x100, where ‘T’ indicates the number of days the MESB treated animals survived and ‘C’ indicates the number of days tumor animals survived [25], [27].

### The Chemopreventive Effect of MESB

For each experiment, out of 10 animals, 5 (group 1) were orally fed with MESB (2 g/kg b.wt) for 20 days prior to the EAC injection and treatment was continued up to 45 days. Group 2 animals were considered as tumor control with no MESB treatment. The tumor volumes were measured after 12 days of EAC injection in both group 1 and group 2 animals. Each experiment was repeated two independent times. The percentage of increase in lifespan was calculated and compared with control animals.

**Figure 7:**
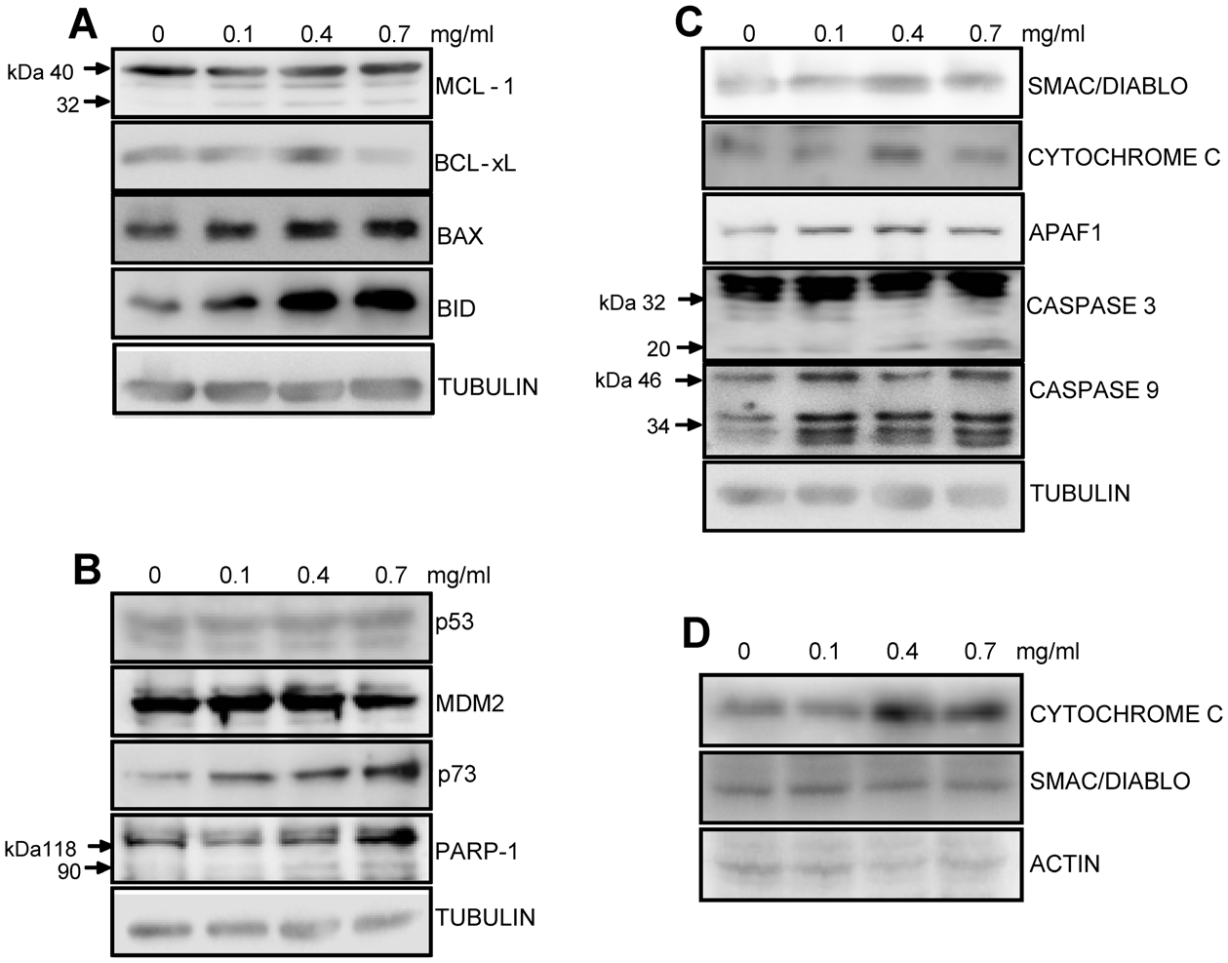
Expression of apoptotic proteins in T47D cells following MESB treatment. Whole cell extracts (**A-C**) and cyotosolic extracts (**D**) were prepared from T47D cells following treatment with MESB (0, 0.1, 0.4, 0.7 mg/ml for 48 h). Western blotting studies were performed using primary antibodies against **(A)** MCL-1, BCL-xL, BAX and BID, **(B)** p53, MDM2, p73 and PARP1, **(C)** SMAC/DIABLO, CYTOCHROME C, APAF1, CASPASE 3 and CASPASE 9, (D) SMAC/DIABLO and CYTOCHROME C. In panels A-C, TUBULIN was used as an internal loading control, while in D, ACTIN was used.

### Histological Evaluation

Tumor and liver tissues of normal and experimental mice were collected and processed as per standard protocol. Briefly, the tissues were embedded in paraffin wax, sectioned at 5–10 µm in rotary microtome (Leica Biosystems, Germany) and stained with hematoxylin and eosin [25], [28]. Each section was evaluated by light microscopy and images were captured (Carl Zeiss, Germany).

### Evaluation of Side Effects in Normal Animals

Swiss albino mice were fed with MESB (2 g/ml) for ten days to assess the side effects. Control and treated groups consisted of 8 mice each. Body weight was measured on every alternate day and the average body weight was plotted. To evaluate the effect of MESB on physiological functions, blood was collected after ten days of MESB treatment and analysed as described earlier [25]. Serum was separated from the blood and used for liver and kidney function tests by comparing the levels of alkaline phosphatase (ALP), creatinine and urea. The blood count was performed by scoring the number of RBC and WBC in whole blood as described earlier [25]. Values obtained were presented as mean±SEM.

**Figure 8:**
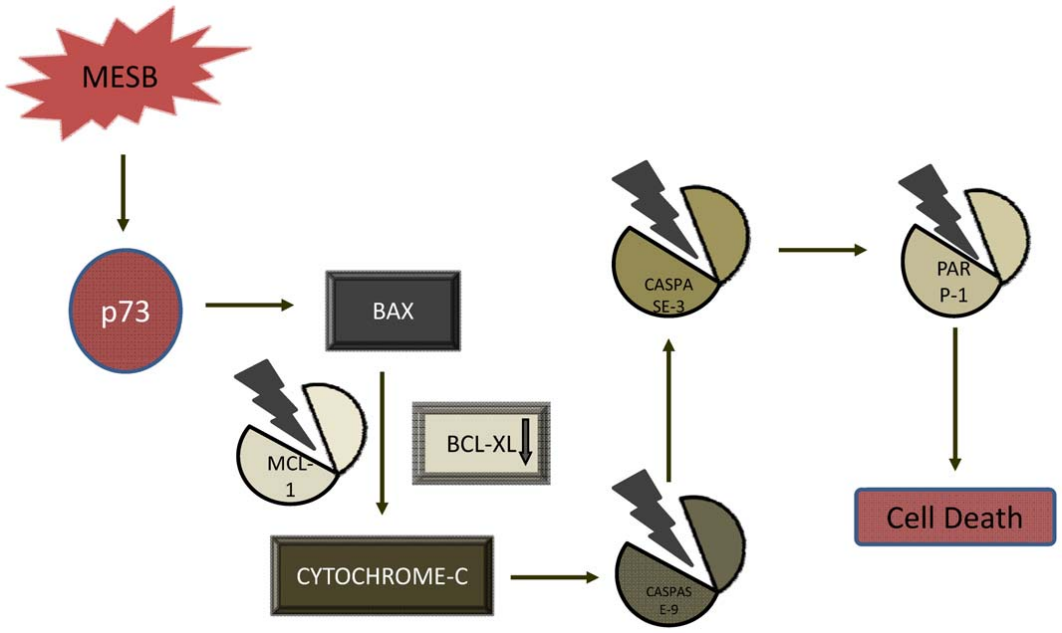
Proposed model for mechanism of MESB induced cytotoxicity. MESB treatment resulted in activation of intrinsic pathway of apoptosis. This is mediated through activation of p73. This activation leads to changes in the level of mitochondrial apoptotic protein, BAX. This may result in the imbalance of proapoptotic/antiapoptotic proteins. The activation of BAX, further leads to cleavage of MCL-1 and release of CYTOCHROME C, which along with APAF1 helps in cleavage of CASPASE 9. Cleaved CASPASE 9 activates CASPASE 3 which further initiates PARP1 cleavage and cell death.

### Immunohistochemical (IHC) Analysis

Immunohistochemical staining was performed on formalin fixed, paraffin embedded tissues, which were sectioned at a thickness of 5 µm. Slides were de-paraffinized using xylene, rehydrated and treated with 3% H_2_O_2_ in PBS and IHC staining was performed as described earlier [29], [30]. In brief, antigen retrieval was done using 0.01 M sodium-citrate buffer (pH 6.0) followed by blocking in PBST containing 0.1% BSA. Primary antibody incubation (Ki67, t-BID or p53BP1; 1∶100) was carried out overnight at 4°C. Slides were washed and incubated with biotinylated secondary antibody (1 h, RT, 1∶200) followed by streptavidin-HRP (1∶1000). Slides were washed (PBS containing 0.1% Tween 20), colour was developed using DAB+H_2_O_2_, counterstained with haematoxylin and mounted in DPX (Sigma-Aldrich, USA). Images were captured using light microscope (Carl Zeiss, Germany).

### Statistical Analysis

Values are expressed as mean ± SEM for control and experimental samples and statistical analysis was performed using one-way ANOVA followed by Dunnett’s test. For this analysis, Graphpad software prism 5.1 was used. The values were considered as statistically significant, if the p-value was equal to or less than 0.05.

## Results

### MESB causes a Time-dependent Cytotoxicity of CEM and T47D Cell Lines

We have evaluated the cytotoxic effect of MESB on human breast cancer (T47D) and leukemia (CEM) cells using trypan blue dye exclusion and MTT assays. Results showed a dose-dependent effect on cell viability of CEM upon treatment with increasing concentrations of MESB (Fig. 1A). Comparable results were also obtained when MTT assay was used to determine the effect of MESB on proliferation of CEM cells (Fig. 1B). The treatment of MESB on T47D cell lines also resulted in detectable reduction in cell viability, particularly at 72 h (Fig. 1D). A similar result was obtained when effect of MESB on cell proliferation was analysed by MTT assay (Fig. 1E). Thus, our results suggest that extracts prepared from strawberry were capable of inducing cytotoxicity in two different cancer cell lines of hematopoietic (leukemia) and epithelial origin (breast cancer).

LDH assay was used to determine the cellular integrity following MESB treatment (0.1, 0.2, 0.5 and 1 mg/ml). Results showed a concentration and time-dependent increase in the LDH release upon treatment with MESB in both CEM and T47D cell lines (Fig. 1C and 1F).

### MESB Treatment Prevents the Progression of Breast Adenocarcinoma in Mice and Results in Increased Life Span

Breast adenocarcinoma cells were used for generating solid tumor in Swiss albino mice. Upon treatment with MESB (from 12^th^ day of tumor development on every alternate day until 45^th^ day, 2 g/kg), a significant reduction in the tumor volume was observed compared to untreated control animals bearing tumor (Fig. 2A, 3A). By 45^th^ day of treatment, most of the MESB treated animals showed no tumor, unlike untreated tumor animals (Fig. 2A and Fig. 3B). More importantly, we observed a significant increase in the lifespan of MESB treated animals (Fig. 2B).

When chemopreventive effect of MESB was studied on tumors induced by breast adenocarcinoma cells, following oral feeding of MESB for 20 days prior to injection of tumor inducing cells, results showed a significant reduction in solid tumor formation as compared to controls (Fig. 2C). Further, we observed a significant increase in the life span of MESB pretreated animals as compared to control group of animals (Fig. 2D). These results indicate that strawberry extracts can provide significant chemoprevention in mice.

Gross anatomical appearance of thigh tissue containing tumor, liver and spleen of control and experimental animals on 30^th^ and 45^th^ day after tumor development further confirmed the effect of MESB in regression of tumor (Fig. 3A, B). The appearance of the treated animals after 45 days as well as morphology of their dissected organs were comparable with those of normal animals indicating that MESB treatment did not lead to visible alterations (Fig. 3).

Histopathological studies were performed on sections from thigh or thigh bearing tumor and liver tissues of normal, tumor bearing and MESB treated animals after 30^th^ and 45^th^ days of treatment using haematoxylin-eosin staining (Fig. 4). Thigh tissue from tumor bearing mouse showed damages in muscle architecture and tumor cell proliferation with very high nuclear staining [Fig. 4A(a–d), B(a–d)]. After treatment with MESB, damages in muscle architecture and tumor cell proliferation were limited indicating the reduction in tumor growth [Fig. 4A(e, f), B(e, f)]. The adverse effect of MESB treatment on other tissues was analysed by taking liver as a model organ. Studies using hematoxylin and eosin stained liver sections showed infiltration of inflammatory cells in animals bearing tumors compared to no tumor controls [Fig. 4C(a-d), D(a–d)]. However, upon treatment with MESB, the liver exhibited mostly normal morphology, with no or limited infiltration in hepatocytes [Fig. 4C(e,f), D(e,f)]. Therefore, the above results suggest that treatment with strawberry fruit crude extracts did not adversely affect the morphology, anatomy or physiology of the other organs.

In order to evaluate side effects of MESB, normal mice were fed with MESB for 10 days and results showed similar levels of serum profile (alkaline phosphatase, creatinine and urea) compared to untreated controls (Fig. 5B). Further there was no significant difference in RBC and WBC counts in MESB treated mice compared to the controls (Fig. 5B). Besides, there was no significant change in body weight measured after 10 days of MESB treatment (Fig. 5A).

### Effect of MESB Treatment on the Expression of Ki67, p53BP1, BID and t-BID in Tumor Tissues

Ki67 is a cell proliferation marker for tumor progression [31]. Immunohistochemical staining of Ki67 protein tumor section showed increased cell proliferation in untreated animals bearing tumor, while it decreased upon treatment with MESB (Fig. 6A). An enhanced expression of p53 binding protein 1(p53BP1), a DNA damage sensor, was observed upon treatment with MESB (Fig. 6B). We have also observed activation of proapoptotic proteins, BID and t-BID following treatment with MESB compared to untreated tumor tissues (Fig. 6C and D) suggesting the induction of apoptosis in tumor cells in mice. Therefore, our results suggest that MESB treatment inhibits the proliferation of tumor cells by activating apoptosis in mice bearing breast adenocarcinoma allograft.

### MESB Activates Intrinsic Pathway of Apoptosis in Breast Cancer Cells

In order to understand the mechanism by which MESB induces cell death, we chose the breast cancer cell line, T47D, for further investigation. T47D cells were treated with increasing concentrations of MESB, cell extracts were prepared and used for immunoblotting analysis. Results showed activation of apoptotic marker, MCL-1, which acts as a proapoptotic protein upon cleavage. We find that MESB treatment resulted in prominent cleavage of MCL-1 as compared to the control (Fig. 7A). MESB treatment also resulted in downregulation of BCL-xL, an antiapoptotic protein, at the highest concentration studied (Fig. 7A). Results also showed a significant upregulation of expression of proapoptotic proteins such as BAX and BID (Fig. 7A).

Previously, it has been shown that the tumor suppressor gene, p53, is mutated in T47D cells [32], [33]. Consistent to this, we could not find any significant change in p53 expression in this cell line, even upon addition of MESB (Fig. 7B). MDM2 is a modulator of p53 and we observed no considerable difference in its expression when treated with MESB (Fig. 7B). Interestingly in case of p73, a paralogue of p53, we observed a dose-dependent increase in expression (Fig. 7B and 8).

p73 can induce apoptosis through both intrinsic as well as extrinsic pathways [34]. Results showed a low level of PARP cleavage and activation of CASPASE 3 and CASPASE 9 indicating the activation of intrinsic pathway of apoptosis (Fig. 7B, C). A significant increase in the expression of SMAC/DIABLO, CYTOCHROME C and APAF1 upon treatment with MESB as compared to control, also confirmed activation of the intrinsic pathway of apoptosis (Fig. 7C). More importantly, western blotting using cytosolic fractions of MESB treated T47D cells, showed release of CYTOCROME C into the cytosol (Fig. 7D). However, the expression of SMAC/DIABLO did not change significantly (Fig. 7D). Thus, our results suggest that extracts prepared from strawberry fruits induce intrinsic pathway of apoptosis in breast cancer cells (Fig. 8).

## Discussion

The correlation between higher intake of fruits, vegetables and decreased risk of developing certain cancers attributes to the content of antioxidants and other secondary metabolites present in plants. In the current study, we have evaluated the anticancer property of crude extract of strawberry fruits. Our studies using two cell lines of different origin suggest that irrespective of the cancer type, MESB could induce cytotoxicity, as shown by three independent assaying methods. We have used a mice model bearing breast adenocarcinoma to evaluate the chemotherapeutic and chemopreventive potential of strawberry. Besides, we have also identified the mechanism by which the strawberry extracts induce apoptosis in cancer cells.

Previously, it has been reported that strawberry extracts induce cytotoxicity in microgram range [3] whereas, in the present study it was in milligram range. This difference could be attributed to the enrichment of polyphenolic compounds by the removal of free sugars, organic acids, vitamin C etc [3]. Moreover, when enriched polyphenolic compounds were used, normal cells also showed equal sensitivity as cancer cells. In contrast, when methanolic extracts of strawberry were used, we did not observe any toxic effect in normal tissues or side effects in normal mice. This suggests that removal of certain components from strawberry extracts, could affect its specificity, resulting in toxicity to even normal cells.

p53 is a widely studied tumor suppressor gene. In response to DNA damage, p53 can act as a negative regulator for cell survival. It has been shown that most of the tumor malignancies are associated with mutation or allelic loss of p53 gene. In contrast, p73, a protein of p53 family, which shares significant structural and functional similarity with p53 protein, is rarely mutated in cancers [19]. Previously, it has also been reported that p73 isoforms can interact and activate the p53 responsive genes, and can regulate the cell survival or cell death [35]. Therefore, one of the important strategies to induce apoptosis in tumor cells could be by using molecules or extracts that can help in upregulation of p73 [36]. Interestingly in our study, we do find a concentration dependent increase in the expression of p73 upon addition of strawberry extracts in T47D cells, in which p53 function is abrogated. Consistent to this, we observed a dose dependent increase in both p73 and BAX. The increased expression of BAX can result in its penetration to mitochondrial outer membrane leading to release of CYTOCHROME C, thereby activating CASPASE 9 (Fig. 8). Western blot analysis following treatment with MESB indeed confirmed such a hypothesis. Expression of SMAC/DIABLO, a mitochondrial protein that can abolish the inhibitory activity of IAP (inhibitor of apoptotic proteins), also showed a consistent upregulation, upon MESB treatment [37]. APAF1, one of the members of apoptosome, also showed elevated expression, consistent with the release of CYTOCHROME C. Hence, the above results in conjunction with activation of CASPASE 9, CASPASE 3 and PARP1 demonstrated activation of intrinsic pathway of apoptosis by MESB to induce cell death. These results suggest that activation of p73, when p53 is mutated, is a good strategy to induce apoptosis in cancer cells. Previously, studies using polyphenolic rich *Aronia melanocarpa* juice also showed activation of p73 in p53 null cells [38]. Besides, it has been shown that polyphenols isolated from red vines, known as gallic acid can inhibit the induction and progression of colon cancer in mice models [39]. Apart from this, p73 associated induction of apoptosis was also reported in jurkat cell line, when treated with red vine polyphenols [40]. However, all these studies had only limited scope, as they did not assess their effect on normal cells, unlike the present study.

MESB treatment on mice bearing tumor resulted in significant reduction in tumor volume without affecting the function of other organs along with approximately 4-fold increase in the lifespan. The histological evaluation showed that morphology and cellular architecture of the tissues was unaffected by the MESB treatment. Immunohistochemical studies also confirmed a decrease in cell proliferation as well as activation of apoptosis following treatment with MESB, suggesting regression of tumor in mice models. The observed protection from tumorigenesis upon pretreatment with strawberry extracts emphasizes the importance of strawberry fruits. Although the mechanism by which it exerts the chemoprevention is not clear, it reveals the added value of intake of strawberry fruits. Thus, the present study emphasizes the high therapeutic potential of strawberry. Further, our studies revealed that it can modulate the expression of p73, when p53 is mutated in cancers like breast cancer and can activate the mitochondrial pathway of apoptosis to abrogate cancer cell proliferation. Thus, our study clearly shows that strawberry can act as a good dietary, chemopreventive as well as therapeutic agent.
